# Familism and Family Violence in Mexico

**DOI:** 10.1177/08862605251319009

**Published:** 2025-02-18

**Authors:** Nancy Gutiérrez Olivares, David R. Goyes, Sveinung Sandberg

**Affiliations:** 1 University of Oslo, Norway

**Keywords:** domestic violence, familism, family violence, Latin America, Mexico

## Abstract

Families are integral to the organization of Mexican society. In a context where the State is absent or weak, the family serves as a social safety net and is pivotal for everything from housing to paid work. As the structural backbone of Mexican society, the family exists within a widespread cultural representation denominated as *familism*, often characterized by a culture of conflict avoidance, tight relationships, mutual support, and self-sacrifice for the well-being of the family. In other words, the family contributes to a more harmonious society. But organizing society around the family also has a shadow side. Using data from repeat interviews with 50 incarcerated persons in Mexico, we show how family dynamics and the associated culture of familism are tied to abusive domestic relationships—phenomena that are critical to understanding family violence in Mexico. We explore the many links between familism and family violence by an in-depth look at four closely intertwined familism processes that facilitate victimization: preventing victims from disclosing family violence; preventing the family from denouncing violence against one of its members; the victim remaining with the family despite the abuse; and the victim being forced to remain in abusive relationships. These four ways that family structures play into victimization within the family are not exclusive to Mexico or other countries shaped historically by familism, but the cultural and discursive structures of familism amplify them.

## Introduction

“My words, when talking about the house, crack,” wrote Mexican Nobel laureate Paz (1980) in “Pasado en Claro” (A Draft of Shadows), an allegorical poem about his family. “Rooms and rooms inhabited only by their ghosts,” he continued, “only by the rancor of the elderly inhabited. Families, breeding grounds for scorpions.” In this poem, Paz comes to terms with his family, highlighting not only its centrality in his life but also his discomfort with it (Solís, 2021). In an earlier work, Paz (1950) argued that marriage and the family were the basic structure of Mexican society, ensuring stability for its members. He warned that critiquing the family would amount to dissolving the very foundations of society. Paz, and many others, saw the family as the backbone of society, as the community’s “main shared value” (González, 2017, p. 21). Yet, in 1980, in the lines of his poem, Paz exposed the harms wrought by families.

Statistics demonstrate that the family, as Paz poeticized, is at the core of Mexican society. Over 87% of the population lives in shared households, with less than 13% living in single-person households. Of those sharing accommodation, 99% live under the same roof as a kinship-related nuclear and extended family (Censo Nacional de Población y Vivienda, 2020; Encuesta Nacional de Ingresos y Gastos de los Hogares [ENIGH], 2018). Sharing households in Mexico is often an economic necessity, but it also has a strong cultural foundation and ultimately ties the families closer together. The contrast with Western countries is significant: in Europe, for instance, out of 220 million households, around 33% comprise single adults (EUROSTAT, 2018).

Scholars capture the centrality of the family in Mexico with the term *familism*. It encapsulates how societies, in their narratives, task the family with avoiding internal conflicts, maintaining familial relationships, providing support for its members, and teaching self-sacrifice for the benefit of the collective (Ingoldsby, 1991; Keefe, 1984; Sabogal et al., 1987). While decades of research in criminology have shown that close connections within a family can serve as a protective barrier against engagement with criminalized activities (Sommers et al., 1994) and that the family plays a crucial role in desistance (Haas et al., 2004; Ttofi et al., 2012; Zane & Welsh, 2019), explorations of familism have also revealed a shadow side of the family and the culture that positions it as a central societal institution (Fuchsel, 2013).

Given the emphasis in criminology on how families and familism protect family members from involvement in crime, we explore this shadow side or the processes through which familism facilitates family violence. Based on the life stories of 50 people convicted for a variety of crimes and interviewed 3 times in prison, we emphasize how specific family structures and a culture of familism make people more vulnerable to domestic abuse. Our qualitative perspective helps us explain four processes through which familism facilitates victimization. These processes are not unique to Mexico or other countries with strong cultures of familism. However, we argue that familism reinforces these phenomena and that family violence is better understood when viewed in the context of prevailing cultural structures.

## Familism and Family Violence

Family violence is violence that is committed within the family, by one family member toward another. Family violence thus encompasses, but is broader than domestic violence, as the latter usually refers to instances of abuse of intimate partners (see Tolan et al., 2006, for a discussion of these concepts). In family violence, “harm is purposely inflected by those who are supposed to care for or depend on one another” (Tolan et al., 2006, p. 559). In their endeavor to understand the phenomenon, family violence researchers have used a set of theories ranging from individualistic rational choice and biosocial perspectives to sociological functionalist approaches (Gelles, 1985). Yet, as in criminology where it is firmly established that the family fundamentally shapes not only law-abiding but also criminalized behavior (Blumstein et al., 1986; Farrington et al., 2019; Haas et al., 2004), less attention has been given to the impact of *cultural representations* of the family on family violence.

The *sociocultural approach* is a theoretical strand of family violence studies that accounts for the role of cultural elements in the phenomenon. In this line of research, cultures harbor direct and indirect attitudes and norms that condone the use of violence within family relations (Gelles, 1998; Straus et al., 2006). Four specific hypotheses are used in the sociocultural approach to explaining family violence from micro to macro levels: family socialization into violence, a subculture of violence, cultural spillover, and cultural consistency (Levinson, 1988). The cultural consistency hypothesis is particularly relevant for this study in that it encompasses dominant cultural narratives, positing that “family violence is a reflection of basic values that shape norms governing family life, conflict resolution, child-rearing practices, and so forth” (Levinson, 1988, p. 447).

Both in sociocultural approaches within family violence studies and in criminology—fields with significant overlap—most studies that connect social representations of the family with family violence draw on the concept of *familism*. Keefe (1984) describes familism as a value and a behavioral system that stresses cohesiveness and solid intergenerational family ties, providing a strong sense of community support. Familist core values are marked by a deep attachment of individuals to their nuclear and extended families, the interdependence of family members, intense feelings of familial identification, and reciprocity, mutual care, protection, and solidarity among family members (Sabogal et al., 1987).

Familism has been variously defined as “a belief in the sanctity and respect of familial relationships” (Pabon, 1998, p. 3), “the belief that the family and the family’s name and reputation are of the utmost importance” (Curry et al., 2018, p. 176), and an ideology in which “family interests take priority over the individual or any other collective interest” (Poppi & Ardila, 2023, p. 404; see also Fuchsel, 2013; McCluskey & Tovar, 2003). These three elements—the family’s sanctity, the respect of familial relations, and the priority of the family’s interests over individual achievements—have underpinned most criminological investigations on how cultural representations of the family relate to crime. However, not all researchers agree that familism reduces crime and victimization, and research on the topic has produced ambiguous and contradictory findings.

Studies diverge regarding familism’s role in family victimization, describing it variously as positive, ambiguous, or negative. In some research, familism is seen as a defense against family victimization. Curry et al. (2018) argue that familism is a protective factor against domestic violence because members try to keep the family together and protect its sanctity. Messner et al. (2007) state that familism indirectly protects family members from abuse because it leads to family members spending time with each other—a safer behavior than being alone. Familism has also been seen as promoting “guardianship and supervision” and offering “the portfolio of strengths required to . . . break the cycle of violence” (Kuper, 2023, p. 3).

These studies have, however, been challenged or at least qualified. Zavala et al. (2023) describe familism as an “insignificant factor” in protecting women against dating violence. Guerra et al. (2024, pp. 5−6) argued that while familism has been seen as enabling “informal control processes that serve as a shield against delinquent peers and other negative influences,” thereby lowering the “odds of exposure to victimization,” familism has no significant impact on protecting family members from abuse within the family. Alcalde (2010, p. 51) similarly states that the role of familism in victimization is ambiguous: It can increase women’s victimization by “promot[ing] the subordination of women’s interests and needs as well as privileg[ing] men’s authority and power in the family.” But because motherhood is a central value and priority in familism, it can also enable women to leave abusive relationships when domestic violence threatens motherhood. In a meta-analysis, Montenegro et al. (2023, p. 11) found that familism can both “encourage the subordination of women within hierarchical family structures, potentially increasing the risk of SVV [sexual violence victimization] against women” *and* provide social support to victims of sexual violence.

A series of other studies fault familism for facilitating family violence. Fuchsel’s (2013) meta-study found that familism paves the way for sexual abuse because victims are ashamed and afraid of denouncing the relative and perpetrator, thereby leaving the crime unreported and unimpeded (see also Sandberg et al., 2021). Likewise, Amaya and Gray (2021) explain that familism discourages the victim’s disclosure of sexual assault for fear of the adverse reactions of family members (see also Timblin & Hassija, 2023). Another meta-analysis by Green et al. (2024) concluded that familism, often present in honor-based societies, disincentivizes victims to seek help from social services because it is seen as shameful. These studies see familism as contributing to family violence because it subordinates victims to family structures and prevents their disclosure and reporting of abuse. In summary, like many other cultural phenomena, familism can either increase or decrease harm depending on the circumstances.

Familism is present, to varying degrees, in all cultures, but in Mexico, familism is a crucial cultural characteristic. Familist values merge with and permeate cultural discourses embedded in social structures, including political institutions and criminal justice systems (Keefe, 1984; Marti & Cid, 2015; Moore & Cuéllar, 1970; Streit et al., 2018). While family structures have undergone various transformations in the past two decades, including urbanization (Chávez & Pérez-Santillán, 2023; Instituto Nacional de Estadística y Geografía, 2020), changing family composition with an increase in divorce and a decrease in births (Capulín et al., 2016; ENIGH, 2018), and an increasing number of female-led households, the result of social development programs in the early 2000s (Consejo Nacional de Evaluación de la Política de Desarrollo Social, 2019), the family remains central in Mexican society as the foremost institution for domestic problem-solving and ensuring the safety of its members (Capulín et al., 2016; González, 2017).

Dominant cultural narratives, such as familism, are closely intertwined with material and organizational conditions in a dynamic in which social narratives and socio-economic structures continuously reproduce each other. For example, a society based on the family as a security net and the centerpiece of labor and social life depends upon dominant cultural narratives about family. Simultaneously, cultural narratives emerge from the practices they idealize. In Mexico, as in many countries in the Global South, the relative absence or failure of the state makes families the primary safety network for many people. The lack of access to welfare services to cover basic needs forces many to seek out family support (Castillo Fernández & Arzate Salgado, 2016). The family as the problem-solving unit closely ties its members together into extensive networks, establishes reciprocal, de facto, rights (Keefe et al., 1979), and makes members highly dependent on one another, which has both positive and negative outcomes. Family disruptions, for instance, become more significant for those affected because state services are not there to help the family with the disruption or, in case of need, as a means for members to escape the family (Labra, 2011). As such, in Mexico, narratives emerging from familism inform gender roles, caregiving practices, and conflict resolution strategies while embedded in these same practices and organized social life.

In this study, we probe into how familism—as a dominating discourse that is reinforced through family practices—is connected to family violence. We acknowledge the many positive aspects of family structures: thwarting victimization, aiding with deterrence and desistance, and strengthening central social roles where the state is absent or fragile. However, since the role that families and familism play in victimization within the family has received less attention in the literature, it is our focus.

## Methods

From March to July 2022, as part of a large research project on crime in Latin America (CRIMLA), 50 individuals incarcerated in 3 Mexican prisons were interviewed, 43 by a research assistant and 7 by the third author of this article. The participants were selected based on the type of offense they were sentenced for: drug trafficking, kidnapping, murder, sexual crimes, and violent theft. Most often, the participants had committed several of these offenses and many others for which they had not been sentenced. Participants were selected from prisons located in Mexico City and in the State of Mexico, the region of the country that has the highest number of prisoners (Table 1) (Instituto Nacional de Estadística y Geografía, 2023).

**Table 1.** Study Participants: Age and Gender.

Age Group	Women	Men	Total per Age Group
18–24	0	3	3
25–29	4	5	9
30–34	3	4	7
35–39	8	4	12
40–44	3	3	6
45–49	2	1	3
50–54	1	2	3
55–59	0	3	3
60–64	2	0	2
65+	2	0	2
Total	25	25	50

*Note.* One transgender woman is included in the women column.

Each participant was interviewed three times, with a few days or weeks between sessions. The repeat interviews provided continuity in the relationship between interviewer and participant and allowed for nuances in the accounts (Goyes & Sandberg, 2024). Each session lasted on avarage a little more than an hour, meaning that data include 150 interview sessions and more than 175 hr of recorded data. Although far from being statistically representative, the extensive qualitative data we gathered, combined with previous research experience in this context and field, allowed us to explore the dynamics of family and familism in family violence throughout the life course of incarcerated individuals.

Interviews were based on an extensive guide emphasizing family context, childhood, youth, adulthood, crime, drug use, violence, detention, legal process, life in prison, and perceptions of victimhood. We also made a point of letting participants tell their own stories, and interviewers were free to probe topics of particular interest not covered by the interview guide. The interviews were organized as life-story interviews, covering continuities, changes, and fluctuations between the past to the present in victimization, criminal involvement, and family dynamics. Tagg (1985, p. 163) argues that the advantage of life-story interviews is that they provide access to “personal conceptions of the past and all its stages” and are “readily interpretable.” Kohli (1981) further points out that a description of events in temporal order aids in the interviewing because interviewees typically expect organization and structure to a conversation.

The larger research project that this study was a part of consisted of extensive, life-story interviews with 400 imprisoned people in 7 Latin American countries (for further details see www.crimeinlatinamerica.com). The procedure of selecting participants differed from country to country. Since penitentiaries in Mexico did not provide us with confidential lists of prisoners or grant us access to confidential physical prison archives, participants were identified as part of our fieldwork in prisons and by talking to prisoners and staff. They were selected to represent people convicted of different types of crimes. Participants had a criminal history, often complex family relationships, and came from marginalized backgrounds. Thus, their experiences are not representative of the broader Mexican population. The controlled environment of a prison, like many other atmospheres, can also alter how participants discussed or perceived family roles (Copes et al., 2015). Nonetheless, the analysis of these cases provided insights into the most severe or acute forms of family violence, which can help identify patterns that may not be as visible in the general population.

The analysis of the collected data began with an initial broad coding in NVivo of the entire corpus of interviews, resulting in a codebook containing 255 nodes, of which 52 were primary analytic themes identified by using thematic analysis and constant comparison (Strauss & Corbin, 1990). The codebook served as the baseline for a detailed analytical coding process of the interviews. Data coding was discussed and refined through weekly and monthly meetings in the larger project to ensure inter-coder reliability and trustworthiness. For this study, themes related to family violence were identified mainly through codes labeled “family context” and “episodes of violence.” Since themes are not mutually exclusive, other codes such as “identity” and “psychological trauma” provided additional information for further analysis of family violence in this study. An in-depth reading of these codes and data fragments identified familism as a frame of reference and were systematized in the four processes we describe in the analysis.

Each participant was assigned a pseudonym to be used in this article chosen from common names in the home country of the participant. As part of the larger project in which the current study is embedded, we asked participants how they felt about participating, and many expressed their appreciation for the opportunity to talk openly about their lives (Di Marco & Sandberg, 2023). Participants received a snack in the interview (biscuits, etc.), but there was no other incentive to participate. For those who asked questions about the nature of the research before agreeing to be interviewed, further detailed information was provided, enhancing their consent to participate. Alongside ensuring a high level of data security through TSD (Services for Sensitive Data of the University of Oslo), we invested significant effort in ensuring that the interviews were positive experiences for participants.

All participants received an oral and written explanation of their rights in Spanish and formally agreed to participate. This explanation included the purpose of the project, the range of questions in the interview, participants’ freedom to decide whether to participate, the confidentiality and privacy measures taken by the project, and the lack of concrete benefits and drawbacks of participating. To ensure consent over the course of the interviews, interviewers were instructed to let participants know that they could withdraw from the interviews whenever they wanted (which some did) and also afterward, meaning that we would delete the information they had provided. The research project was hosted by the University of Oslo, Norway. Therefore, we sought and obtained authorization from the Norwegian Centre for Research Data (NSD) to collect and store life stories. We also received permission from the Center for Research and Teaching in Economics, Mexico (Number CEI/0002/1).

## Results

Most of the participants from Mexico were raised in hierarchical families, a structure aligned with familism. In these families, individual members are first and foremost subordinate to the family unit, a phenomenon broadly documented in the literature (see, for instance, Umaña-Taylor et al., 2011). Second, children are subordinate to their elders. And third, women are subordinate to the men in the family. Indeed, gender was a crucial element in our study, as has been shown in the literature about familism (see, for instance, Ulibarri et al., 2009). In our sample, it was mainly women who were victims of family violence.

Emma (age 39) describes the dynamics in her family, illustrating the victimization facilitated by what we argue is a form of subordination shaped by familism:

We come from a small town far from the city, and my mother raised us with customs from there. Women couldn’t leave their house . . . we had to be inside, just watching TV, having coffee or dinner. . . . In that small town and my house, men are the ones in charge, the ones who decide, the ones who manage the money . . . they are a bunch of drunks, womanizers, and violent! . . . Like my dad . . . he used to beat my mom and his children. All the defects a human being may have are those of the men in that town, and we, women, cannot complain.

A key phrase in Emma’s statement is “*cannot* complain.” Such statements do not mean that women *abstain from* complaining but must be read as a critique of what they perceive as the problems of a dominant cultural narrative. The cultural value of supporting and protecting the family, including its men, is connected to a discursive maxim against exposing family violence. We argue that familism and its maxims may facilitate domestic victimization through four closely connected processes: (a) preventing victims from disclosing family violence, (b) preventing the family from denouncing the violence against one of its members, (c) victims remaining with the family despite the abuse, and (d) victims being forced to remain in abusive relationships.

### Preventing Victims from Disclosing Family Violence

Antonia, 34 years old at the time of the interview, was raised by her mother, aunt, and grandmother. When Antonia was a child, she was repeatedly raped by one of her stepfathers. During one such assault, Antonia screamed so loud that her grandfather heard her from the second floor and ran downstairs to find out what was wrong. “He fell down the stairs and died,” Antonia said. “I kept quiet and said nothing because I felt it was my fault.” The word *it* in Antonia’s testimony is ambiguous: It may refer to the rape (as in “it was my fault that I was raped”) or to her grandfather’s death (“it was my fault that he fell and died”). Either way, the fragment reveals a trait of familism, namely, that it is preferable to sacrifice oneself by internalizing guilt than to compromise the family’s cohesion. Antonia dutifully protected the family’s cohesion, considering herself culpable so that other family members would not discover her victimization. Antonia did not disclose the abuse to safeguard “the family peace,” as she called it. Instead, she attempted suicide on several occasions. Antonia only confronted the family when she reached the age of majority: “When I turned eighteen, I got drunk for the first time [laughs]. And well, I blew off steam,” she said, “I confessed everything. But my mother stood up for my stepfather and said that I had offered myself to him.”

Lucero (45) was often hit by one of her uncles during her childhood. She did not disclose him, and nobody noticed the abuse. Her reason for remaining quiet was that “families are very important . . . because the fact that someone offers you their home is a big deal; it is a big responsibility for the person, right?” Lucero eventually fled her home, pushed by the physical violence she was subjected to, but she never revealed what had transpired. She wanted to protect her family. “Families will always want the best for their people,” she said. Monse (39) experienced repeated verbal abuse during childhood. Her mother often called her a bitch, a burden, and a slut. The insults “hurt more than any spank,” Monse said, but the admiration she felt for her mother kept her from exposing the abuse. She was bound by fear and the idea that family is sacred. “I’m going to cause a problem, and that’s going to make my mother hate me even more,” Monse reflected. She had never disclosed the verbal abuse until she was interviewed.

Most abused children, overwhelmed by the fear, shame, and trauma stemming from the violence lack the capability to report an abusive guardian (Stanley et al., 2012). As the cases of Antonia, Lucero, and Monse illustrate, familism may erect a further barrier deterring victims from exposing violence in the family; revealing information that places a family in a bad light leads to embarrassment, guilt, and even the cessation of support for the abused person (as we show next). Indeed, a broadly documented tenet of familism is protecting the family unit (Guilamo-Ramos et al., 2009), which can further blind people to abuse or make it difficult for them to report it when they are aware of it.

### Preventing the Family from Denouncing Violence Against One of Its Members

When Lalo (32) was a child, his stepfather regularly beat him. Lalo’s mother was aware of the violence but covered for it. “My mother didn’t take me to school for about fifteen days,” Lalo said. “She said that I had chickenpox, but I never had chickenpox. The truth is that they started monitoring for violence in my school, and I had bruises all over me.” Lalo’s mother was afraid the teachers would find out about the domestic violence. Trauma and high loyalty to the family made Lalo unable to disclose the violence, and he remained with the family longer than he would have had to. What is different in this case from the first process we analyzed is that even when members of the family—other than the victim and the perpetrator—are aware of the abuse, they hide it to protect the family’s integrity and honor. Lalo only realized as an adult that the reason he spent significant time on the streets as a child was to escape the violence. “As a child, I thought it was because maybe I liked being on the streets with my friends,” he said, “but when you grow up, you analyze the situation and say ‘No, I think it was to seek a bit of peace,’ right?”

Throughout his childhood, Felipe (55) was a victim of his father’s rage. He suffered verbal abuse and constant beatings, and their relationship deteriorated: “I wanted him dead for all the damage he caused me,” Felipe said. Suicidal thoughts were constant for Felipe: “The only reason I didn’t take my life was the strength granny gave me.” Yet, for all the strength Felipe’s grandmother provided, she was aware of the abuse but never reported it to the authorities; she limited herself to treating Felipe’s wounds. Ana (34) was repeatedly raped from age 4 to 10 by her brother. Ana’s brother only stopped the assaults when Ana began menstruating. Her mother knew about the abuse but never acted on this information. In fact, she beat her daughter for letting others find out what transpired at home when schoolteachers reported Ana’s altered behavior (as a consequence of the abuse) to her mother.

Reporting the violence perpetrated by a relative can lead to family sanctions. Gloria’s (47) mother had an alcohol use disorder, so she was raised by several of her uncles. One of them used their closeness to molest and rape her over the course of a year. Gloria’s grandmother discovered the abuse when she was bathing Gloria. “My grandmom took me to the police station,” she told us. “I got several medical checkups and was asked many questions.” And although Gloria’s uncle was arrested, her mother covered for him and signed a letter exonerating him from all responsibility—the criminal process was brought to a halt. Gloria and her grandmother were expelled from the family for exposing the abuse. “Nobody in our family wanted me,” she said. “I felt the resentment from my family for having accused my uncle.”

Lalo, Felipe, Ana, and Gloria had family members that hid the violence perpetrated against them. Other participants were the ones covering up the violence of a family member. Daniel (37) described the ordinariness of violence at home as he narrated his father’s repeated attacks on his mother: “I remember that I was about four years old when my dad struck my mom so hard that I had to ride on his back to make him stop.” Daniel considered his father to be “violent by nature,” but he never, even as an adult, exposed him, and he suspended all moral judgment. “I cannot judge my dad. I’m not the one to do it, right?” he said. “I’ll let God judge him and make him pay for what he did to me, even when I was a child.” Again, the word *cannot* as used by Daniel is a central trope in familism narratives of family violence (Sandberg, 2016): members *cannot* “betray” each other, even if that means being a bystander as a family member is abused. Daniel was 4 years old when he first witnessed violence against his mother and reacted in a way that one would expect at that age, pounding on his father’s back to get him to stop. As he grew older and became an adult, trauma and the values of familism, alongside more general defense mechanisms deterred him from exposing the violence he witnessed at home.

In societies, cultures, and contexts where familism dominates, disrupting a so-called well-integrated family may become more distressing than dealing with conflicts or violence in family relationships (Keefe et al., 1979). In settings shaped by familism, people often see the family as the fundamental unit of survival that keeps members attached to it even if it is destructive. Male perpetrators are usually high on the family’s hierarchy and seen as “respected family members” (Fuchsel, 2013, p. 380), so they are doubly protected by their relatives. Therefore, familism may arguably add to, and strengthen, other processes preventing the family from denouncing violence against one of its members.

### Staying with the Family Despite the Abuse

Jesús (31) never experienced his father’s love, only his rage. Once his father flogged him, and his worried mother had to intervene. “Stop it, enough!” Jesús remembered her mother yelling, “You treat him as if he were not your son.” Imitating their voices, he continued narrating how the episode unfolded, “My dad replied something that I will never forget. I don’t even remember how old I was, I was very young, but I will never forget his words: ‘Yes, bitch! Who did you fuck, bitch? Jesús doesn’t have my [skin] color!’” Reflecting on the first two processes we described, Jesús never revealed his father, and neither did his mother. Further, and this is how process 3 differs from the first two, Jesús lived with them for many years during and after the abuse. Familism entails an unconditional loyalty to the family (Fuchsel, 2013; Rodriguez, 2008), affecting victims’ discernment and making it difficult for them to dislodge themselves from the location of violence. Jesús does not hold a grudge against his father and has even defended him to one of his brothers.

Antonia (34) was raised by her aunt with her mother’s consent immediately after she was born. Her family concealed from her that the woman she knew as her aunt was actually her biological mother. From age 7 to 12, her biological mother’s boyfriend raped her. Still, Antonia remained living with her family, and in her adolescence, she worked alongside her biological mother for a drug trafficking organization. During these years, Antonia showed great loyalty: “My mom is alive thanks to me,” Antonia explained, “the organization was going to kill her . . . but I talked to the boss, and I offered to receive the punishment myself instead of her. . . . I exchanged her life for mine.” In spite of Antonia’s loyalty, Antonia’s biological mother handed her over to the police.

Mónica (44) was repeatedly abused as a child. Once, when she returned home from a friend’s house, her stepfather was waiting for her with a cable: “He beat me up. I even got a bloodshot eye. While he beat me, I looked at my mom like, ‘Aren’t you going to do anything?’ She yelled at me, ‘You misbehaved.’” Mónica stayed at home longer than she would have had to, finding it difficult to distance herself from her abusive family. Gil (24) was mostly alone during his formative years. His mother was always at work, as was his grandmother. “That’s why my brothers and I had a tough life,” he explained. “My uncles were the ones who took care of us, and they were always drunk and beat us.” Those same uncles involved Gil in drug trafficking. His loyalty toward them increased with time, and he fought and injured some of his uncle’s enemies. “It was a conflict I had nothing to do with,” he said, “but I will stand by my family’s side.” And even though his family has never reciprocated his loyalty (“I always have to beg them to visit me”), Gil maintains his unconditional support for his family.

An important trait of familism is the strong bond created between family members, as the cases of Jesús, Mónica, Antonia, and Gil demonstrate. Even for children and teenagers traumatized by family violence in cultures not marked by familism, it is rare for children to detach themselves from their families (Gelles, 1997). However, familism further hinders this by tying abused members even more tightly into violent families as adults, since familism often leads people to prioritize the family “even if the costs of these relationships exceed the benefits” (Singelis et al., 1995, p. 244). Mónica’s reflections during the interview are illustrative; when we spoke with her, she had just come from a prison workshop about empowerment where she sought her mother’s forgiveness for running away from home and not standing by her side.

### Being Forced to Remain in Abusive Relationships

Emma (39) was abused by her partner but tried to keep it hidden from her parents: “I didn’t want my mom and dad seeing how he beat me. I didn’t want to be one more problem at home. I felt it would cause them a headache.” Emma and her partner moved to a room in her mother-in-law’s house, far away from her family: “At least no one was going to see us there,” she said. The situation deteriorated here. Her husband’s use of alcohol and drugs worsened, and he became much more violent to the point of trying to kill Emma and their children on three different occasions. His first attempt was a murder-suicide in which he opened the stove gas valves when they were asleep. Months later, he added rat poison to Emma’s and the children’s food, and some weeks later, he tried to stab them. Emma’s mother-in-law, brothers-in-law, and her mother knew about the murder attempts but neither Emma nor her relatives reported the abuse (processes 1 and 2); Emma stood by her husband’s side throughout significant violence, even when her life was in danger (process 3). When she tried to leave him, her relatives forced her to stay (process 4). “You can’t leave him,” Emma’s mother said. “If I have stayed so many years with your father beating me all the time, you can also do it.”

Gloria’s grandmother ruled the family; she decided on right and wrong. Her family, like most familist units, followed tradition and time-honored customs. Gloria was forced to marry a man handpicked by her grandmother. She was also not allowed to terminate an unwanted pregnancy. Gloria’s husband was violent and unfaithful, yet her grandmother insisted she remain with him. Even though Gloria was critical of the choices her family made for her, she found it difficult to disentangle her life from her family’s dictums. Lila (26), pushed by the extreme poverty brought on by her father’s death, married to relieve her mother’s burden. The couple lived with her husband’s parents, and Lila was forced to work in a coal mine under extreme conditions: “I woke up at 5 in the morning and returned home around 10 or 11 at night,” Lila told us. She turned over all her wages to her husband as the custom of their native town dictated. When Lila tried to return to her mother’s home, her mother rejected her and ordered her to return to her husband. Monica, whose case also illustrates process 3, began a relationship with an imprisoned man and had a son with him. She took advantage of her prison visits to sell food and earn money to support the family, herself, her son, and her husband. Her incarcerated partner, however, was jealous of Monica’s male customers and accused her of “only wanting to work in prison to flirt with other guys.” He often beat Monica out of jealousy. Wanting to escape the situation, Monica, with her son, flew to a distant city. Her mother, who liked Monica’s incarcerated partner because he had money and guns, convinced her to return home without revealing her ulterior motive.

My whole family welcomed me with a party at my mom’s place. During the party, I went to the store to buy a Viña [a vodka-based drink]. When I returned, I heard a song in the background. He [Monica’s incarcerated partner] used to sing it to me. Suddenly, he emerged from behind me and said, “Hello princess, surprise!” I turned around to glare at my mom; I wanted to tell her with my eyes that she screwed up.

These examples show, first, that familism intersects with dominant gendered discourses to reinforce hegemonic masculinity and machismo: men are considered the family authority to whom women are subordinated, and the entire family legitimizes men’s superior status (Alcalde, 2010). Further, and central to the fourth process, is that in instances of domestic violence, familism not only demands the subsumption of the self to protect the family and silent complicity to conceal domestic violence, but it also often strengthens social control in which individuals are coerced into remaining in destructive domestic arrangements for the sake of so-called family harmony.

## Concluding Remarks

Worldwide, people in prison—especially women—have experienced more physical, sexual, and psychological abuse than the general population (Bucerius & Sandberg, 2022; Goyes, 2024; Rodriguez, 2008). We argue that familism, with the values behind the dictum *not* to disclose domestic violence and the mandate not to leave, can be facilitating factors. An overly strong connection to the family and an adherence to the values of familism, which lock in and immobilize people, may enable and further entrench victimization within the family.

Studying the relationship between family violence and a society’s dominant cultural narratives poses numerous methodological challenges: effectively defining culture, determining if representative cases characterize said culture, delineating cultural boundaries, and empirically identifying cross-cultural variations (Gelles & Cornell, 1983). Importantly, not everyone shaped by the same culture reacts in the same way, and familism exists not as a binary trait across cultures, but as a continuum, including both stronger and weaker versions. At the weaker end of the continuum, familism is one of many cultural influences, some even challenging the significance of families, while at the stronger end, familism dominates culture and social life. Mexico’s familism is at the stronger end of the continuum and arguably it is an important factor in understanding family violence.

Familism is not univocally negative—it is a complex cultural discourse with ambiguous societal impact. Some of the studies we reviewed above detail the positive sides of familism, and our data also revealed the prosocial sides of familism. Yet, it became apparent that familism made many of our participants easy prey to family violence and railroaded efforts to expose or escape the abuse. Dominant cultural narratives exert significant influence over people and facilitate victimization by subordinating people to the family unit and in particular to the male members at the top of the hierarchy. Familism also facilitates victimization by preventing people from disclosing and reporting abuse.

Researchers across the decades—including ourselves—have identified familism as one of Mexico’s dominant cultural narratives (Keefe, 1984; Miller, 1994; Moore & Cuéllar, 1970; Patterson & Marsiglia, 2000; Sandberg et al., 2021). In Mexico, to varying degrees, people tend to draw on familism to build their identities and the narratives that shape their lives. The four processes described above show that individuals raised in a culture that reveres the family might prioritize this institution above the individuals in it and see themselves as destined to be in the family. Even when there is violence in a family, the victim often remains loyal to the people who harm them. While this is common in many contexts it might be even more important in societies and cultures shaped by familism.

In our life-story interviews with incarcerated persons in Mexico, we identified four processes within familism that shape victimization in family violence: preventing victims from disclosing family violence, preventing the family from reporting the family violence of one of its members, the victim remaining with the family despite the abuse, and the victim being forced to remain in an abusive relationship. Since familism as a cultural pillar is not unique to Mexico, our study can be helpful in understanding family dynamics in the context of domestic violence in many different societal contexts. The four processes we describe might be especially important for locations where familism dominates culturally and essentially shapes social and economic life.

Our qualitative study of the shadow side of familism is also a reminder of the importance of culture in understanding interpersonal violence—including family violence. Culture in and of itself does not cause violence or crime, but its narratives give shape to it and can heighten, or reduce, the likelihood of such behavior. We are, of course, not the first to say that culture matters. The *cultural consistency hypothesis* (Levinson, 1988), for instance, forefronts the significance of widespread cultural representations for family violence studies. We continue such a tradition of cultural analysis within interpersonal violence studies, adding that culture is not an abstract and vague construct but a recognizable discourse that operates in real-life situations through specific narratives. Capturing such processes and their characteristics increases the precision with which we understand culture, and by extension, its relation to violence. Further, identifying the specific stories and processes through which culture contributes to shaping social interactions necessarily leads to nuanced readings of society. Culture and its narratives are never exclusively positive or negative; instead, they contribute to generating a multitude of outcomes. Sometimes, however, there is a tendency in the way culture shapes certain phenomena.

Our study highlights the importance of culture, identifies the narratives through which it co-shapes social dynamics, and disentangles its role in family violence. The four processes we described illustrate how a dominant cultural narrative, familism, can facilitate family violence. By emphasizing the negative, we nuance the widespread positive readings of familism, thereby illuminating its ambiguous outcomes. Familism, as any other dominant cultural discourse, has a multitude of outcomes that depend on the specific material circumstances in which it is played out. While we steer away from assigning causality to culture and avoid essentializing the culture of specific social groups, we claim, along with many others, that culture and its narratives matter for the study of interpersonal and family violence. In our study, more concretely, we argue that the cultural and discursive structures of familism may serve to amplify the processes involved in family violence.
